# A Method for Accurate Reconstructions of the Upper Airway Using Magnetic Resonance Images

**DOI:** 10.1371/journal.pone.0130186

**Published:** 2015-06-11

**Authors:** Huahui Xiong, Xiaoqing Huang, Yong Li, Jianhong Li, Junfang Xian, Yaqi Huang

**Affiliations:** 1 School of Biomedical Engineering, Capital Medical University, Beijing, China; 2 Beijing Key Laboratory of Fundamental Research on Biomechanics in Clinical Application, Capital Medical University, Beijing, China; 3 Department of Radiology, Beijing Tongren Hospital, Capital Medical University, Beijing, China; Beijing Institiute of Otolaryngology, CHINA

## Abstract

**Objective:**

The purpose of this study is to provide an optimized method to reconstruct the structure of the upper airway (UA) based on magnetic resonance imaging (MRI) that can faithfully show the anatomical structure with a smooth surface without artificial modifications.

**Methods:**

MRI was performed on the head and neck of a healthy young male participant in the axial, coronal and sagittal planes to acquire images of the UA. The level set method was used to segment the boundary of the UA. The boundaries in the three scanning planes were registered according to the positions of crossing points and anatomical characteristics using a Matlab program. Finally, the three-dimensional (3D) NURBS (Non-Uniform Rational B-Splines) surface of the UA was constructed using the registered boundaries in all three different planes.

**Results:**

A smooth 3D structure of the UA was constructed, which captured the anatomical features from the three anatomical planes, particularly the location of the anterior wall of the nasopharynx. The volume and area of every cross section of the UA can be calculated from the constructed 3D model of UA.

**Conclusions:**

A complete scheme of reconstruction of the UA was proposed, which can be used to measure and evaluate the 3D upper airway accurately.

## Introduction

The anatomical structure of the three-dimensional (3D) upper airway (UA) is essential to evaluate and surgically treat the disease of obstructive sleep apnea (OSA) as well as other respiratory disorders [1–5]. 3D UA models have become important tools in investigating the relationship between anatomical structure and the mechanics of respiration in recent years [6–9]. Many Computational Fluid Dynamics (CFD) models based on the structure of the 3D airway are used to study the behavior of airflow and particle deposition to improve the efficiency of drug delivery in the nasal cavity [10,11]. A major challenge in these models is to build a 3D structure of the UA aided by medical image processing software. Most computational models use computed tomography (CT) images to reconstruct the 3D upper airway. For conventional CT images, the difference in gray levels between the airway and the bony boundary is distinct, that decreases the difficulty of segmenting the nasal boundary. However, there are still many challenges in reconstructing the upper airway geometry using CT images because of its complexity in the anatomical structure [12]. Although there is some medical processing software available that can be used to build the 3D UA, questions about the accuracy and validation of the models still exists [13]. Do these 3D models accurately and faithfully reflect the real anatomical structure? Recently, Alsufyani et al. evaluated the upper airway models based on cone beam CT (CBCT) and concluded that there was still a lack of validation and accuracy for these models [14]. In addition to their suggestion on the evaluation of the model accuracy using statistical methods, developing a new method that can faithfully build the complex anatomical structure of the UA and overcome the limitation of CT images is still a big challenge.

The anatomical structure of the UA is relevant to understanding respiration function. For example, the narrow space of the nasal cavity facilitates the functions of the nose, such as filtering and humidifying the air, and making olfaction efficacious [15]. This anatomical feature of the UA provides a great obstacle for the automatic segmentation of the UA boundary. Meanwhile, previous studies have found that a small difference in velopharynx boundary shape between sleep apnea patients and controls could lead to significant difference in intraluminal pressures [16]. These results suggest that the airflow in the upper airway is sensitive to the geometrical structure of the UA. Thus, one must be careful when modeling the UA to keep its structure as close to the real structure of the UA as possible.

Images obtained using magnetic resonance imaging (MRI) method have superior soft tissue contrast. As far as we know, to date, there are far fewer UA models built based on MRI images than CT images [17–23]. The reason is not only that the scanning time of CT is much shorter than that of MRI, but also CT images can produce better resolution for bony tissue. In addition, the cost of CT scanning is relatively low compared with MRI. However, MRI scanning is safer since it contains no dangerous X-ray radiation, which cannot be ignored when taking CT scans. One more important point is that MRI has much higher resolution for soft tissues. We know that the UA is enclosed not only by bony tissues but also soft tissues, such as the soft palate and tongue, etc. Furthermore, the nasal cavity consists of bone and cartilage, covered by the mucous membrane. Therefore, MRI can significantly improve accuracy in recognizing the boundary locations of the UA [24,25]. Thus, MRI is a better candidate for clearly showing the structure of the UA.

The level set method has been used to segment the images [26–28]. Compared with other methods, such as the threshold method or the differential operator, the level set method works better at dealing with tissues with complex boundaries. To make use of its excellent topology deformation and decrease the adverse effects of inhomogeneities of image intensity, it needs to invoke manual operation to choose the initial region for the evolution function of the level set method.

Most current ways of constructing UA models use images from only one scanning direction, but using images from all three mutually perpendicular planes, the axial, sagittal, and coronal planes, can provide more information about the anatomical features of the UA. The NURBS (Non-Uniform Rational B-Spline) surface can be used to convert the segmented two-dimensional (2D) boundaries into a 3D anatomical structure. The advantage of the NURBS surface is its smoothness and good adaption to complex surfaces [29, 30].

In the presented study, we improve the method of the 3D reconstruction in three aspects: 1) For the segmentation of the UA boundary, the level set method is modified by manual operation to segment the UA boundaries effectively; 2) Based on MRI images from the three orthogonal scanning planes, the complementary anatomical information is provided through the process of registration of the boundaries in different scanning planes; 3) The reconstructed smoothing surface of the UA using the NURBS surface naturally coincides with the segmented 2D boundaries without artificial modification. Under such considerations, we propose an anatomically accurate scheme to construct the 3D UA, which can reduce the arbitrariness and preserve the anatomical features of the UA during the process of reconstruction.

## Methods and Materials

### MRI protocols

MRI slices of the nasal cavity and oropharynx region of a young male participant were taken in the supine position. The MR sequence was chosen as T2-FRFSE. The scanning was conducted successively at three orthogonal orientations (axial, sagittal and coronal plane) in the region of the UA. The axial slices were acquired with a field of view of 22×22 cm from top of the nasal cavity to the epiglottis, with 2.5 mm thickness and a 0.5 mm gap between slices. Then, the sagittal scans were performed with a field of view of 28×28 cm in the range from the external lateral wall of the left maxillary sinus to the external lateral wall of the right maxillary sinus, with 2.5 mm thickness and a 0.5 mm gap between slices. Last, the coronal slices were acquired with a field of view of 24×24 cm in the region from the tip of the nose to the posterior portion of the cervical vertebrae, with 2.5 mm thickness and a 1.5 mm gap between slices. All the images in the three orientations were obtained with a matrix of 512×512 pixels.

Spin-echo MRI of the upper airway was performed during wakefulness using a GE Signa HDxt 3.0T MRI scanner in Beijing Tongren Hospital, China. The total scan time was about 30 minutes.

This study was approved by the Ethics Committee of the Capital Medical University, Beijing, China. The participant signed a consent form prior to participation.

### Image Processing

**Fig 1:**
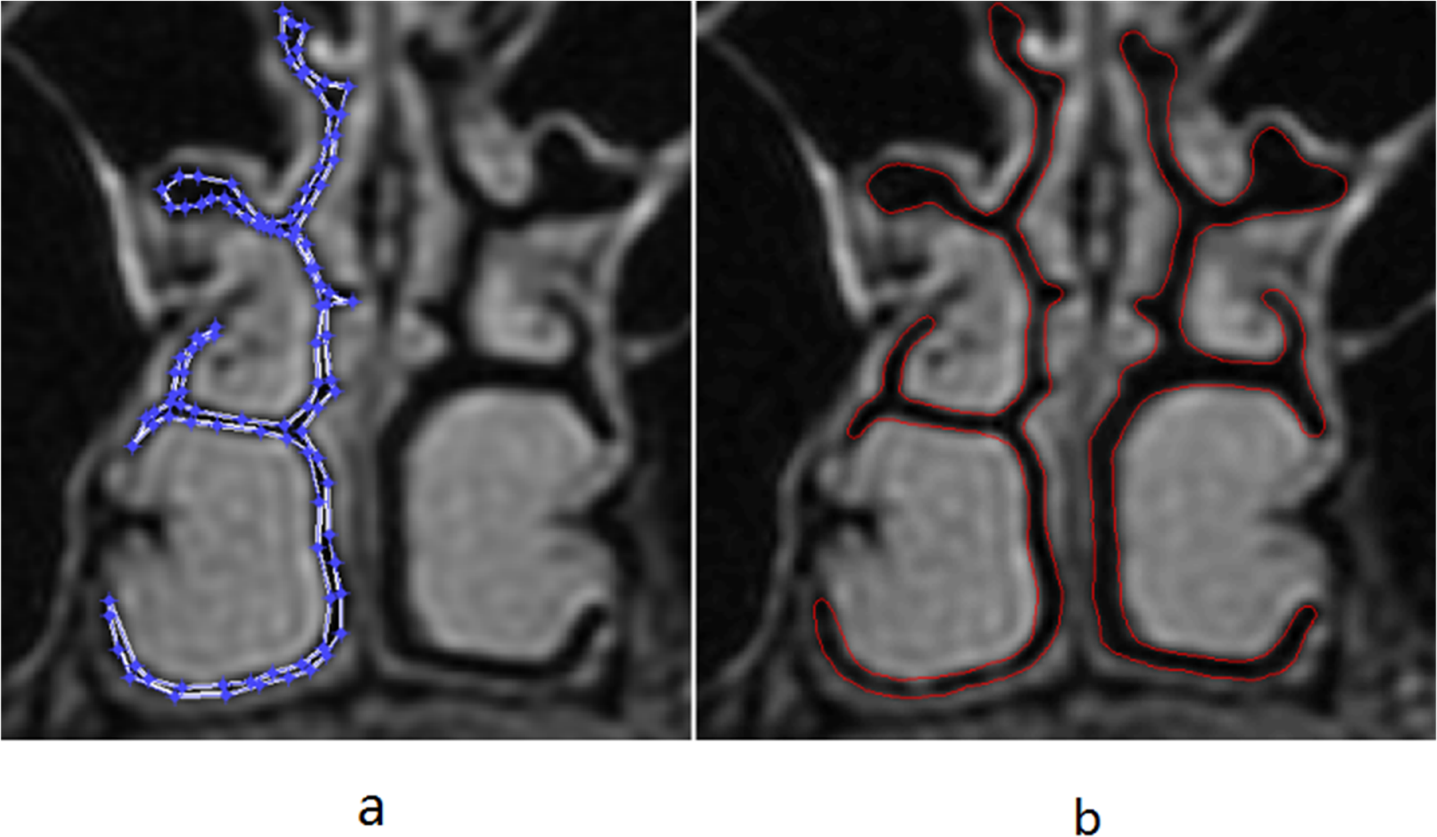
The process of segmentation of the nasal boundary. a) The coronal image of the nasal cavity with the initial region of manual selection. b) The segmented boundary of the nasal cavity obtained by the level set method based on the initial region in the left panel.

All the DICOM images were transformed to the BitMap format using the free software program RadiAnt DICOM Viewer for the following image processing. Then, the level set method was used to segment the 2D boundary of the UA. The boundaries were marked using the zero value of the evolution function distributed in the region of 2D image. This characteristic ensures that the evolution curve of the level set method has the power of arbitrary deformation. The evolution curve ends at the desired boundary when the constructed energy function achieves an extreme value. For the narrow cavity, it is difficult to use the level set method directly because the nasal meatus is curving and quite cramped. This makes it hard to specify the inner part of the nasal meatus as the initial region for the evolution function. We then cut the whole region of the nasal cavity in the coronal images and amplified the selected region up to three times. Since the quality of segmentation depends on the shape and location of the prescribed initial region, a better way is to manually specify the initial boundary line. Thus, the points that enclose the region as the given initial zone are manually marked. To obtain the object boundary, the region of the nasal cavity should be chosen as the initial region such that the final position of the evolution curve could approach the outline of the nasal cavity. The final boundary is obtained when the evolution curve stopped moving. Fig 1 shows the process of segmentation of the nasal boundary. As shown in Fig 1B, the segmented boundary coincides well with the real boundary of the nasal cavity, which indicates that the level set method, aided with manual operation, can effectively handle the complex structure of the nasal cavity.

**Fig 2:**
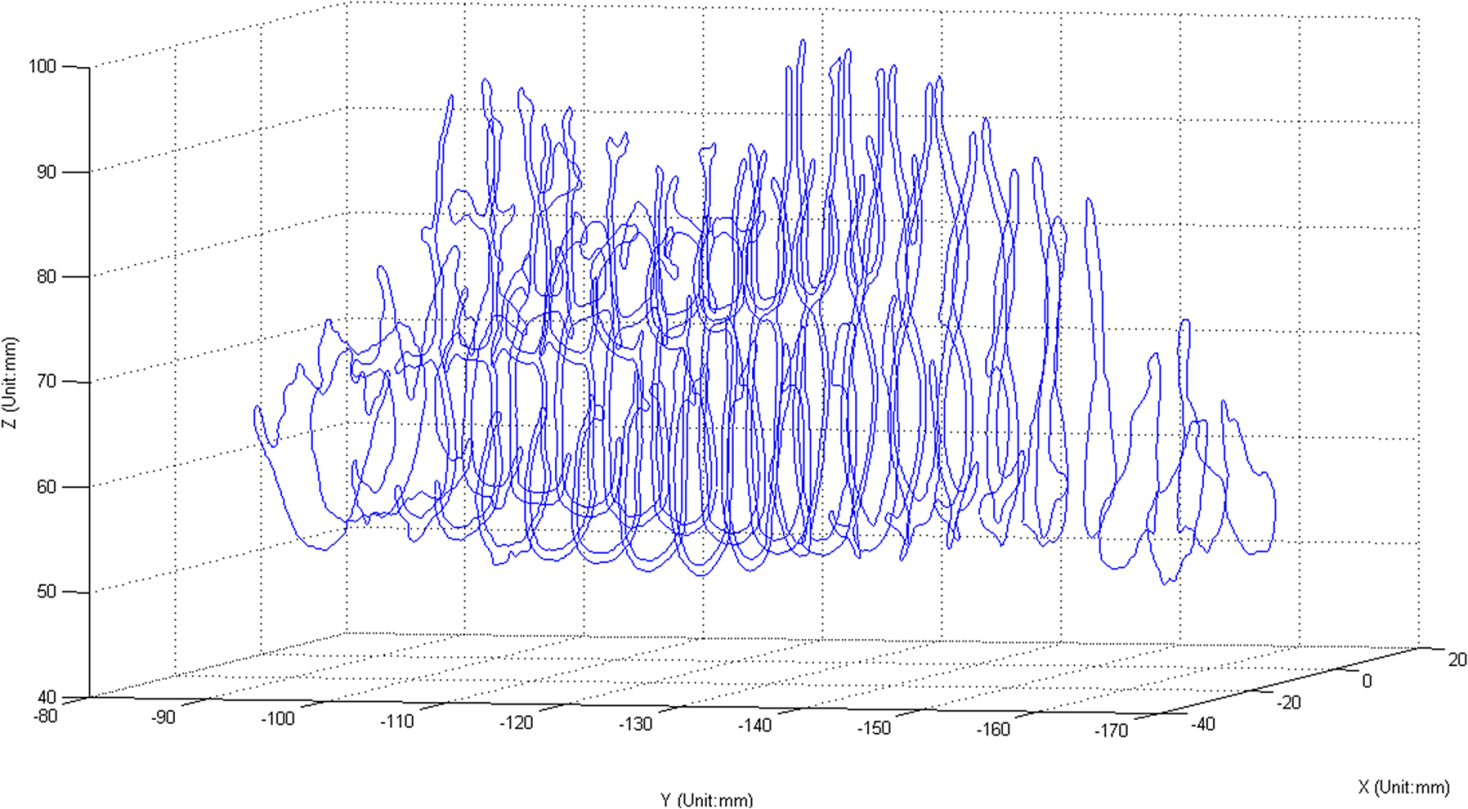
The boundaries of the nasal cavity in the coronal section. All the boundaries of the nasal cavity in coronal plane are plotted from the naris to the nasopharynx. The distance between the neighboring curves is 4 mm.

**Fig 3:**
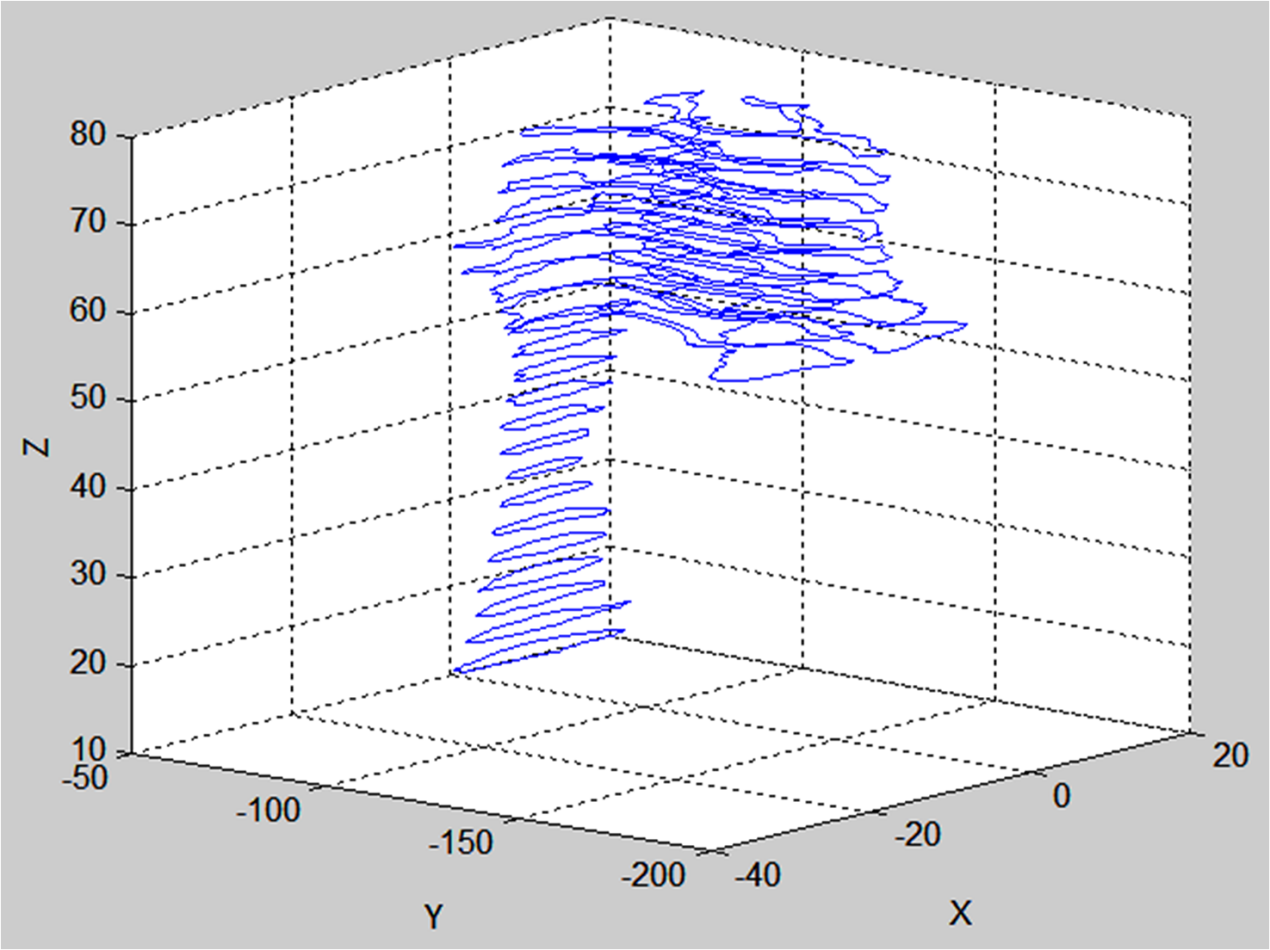
The axial boundaries from the nasal cavity to the oropharynx. The distance between the neighboring curves is 3 mm.

For each slice, the coordinates of the boundary points obtained using the level set method were output to a text file. Fig 2 displays the boundaries of the nasal cavity in the coronal section from the vestibular region to the nasopharynx. In the same way, the axial boundaries of the UA were obtained and are shown in Fig 3. The sagittal boundaries of the UA serve to calibrate the reconstructed structure for both the nasal cavity and the oropharynx.

### Image Registration

At this point, we have obtained the boundaries of the upper airway in three orthogonal orientations. As a result of the partial volume effect and the movement of the head of the participant during the scanning process, the segmented boundaries in different orientations may not coincide with each other. For example, the boundaries in the coronal plane may not have the same crossing points with the boundaries in the axial plane for the same region of the UA. Sometimes, the deviation between the corresponding crossing points is pronounced. Therefore, before reconstruction, one necessary task is to register the segmented boundaries between the differently-oriented planes.

For the nasal cavity, the coronal plane images have better resolution for the warped and narrow airway compared with images in the sagittal and axial planes, We mainly use the coronal images to build the nasal cavity and nasopharynx, together with a few axial and sagittal images to calibrate the geometry structure. The axial boundaries are also used to construct the oropharynx. Thus, in registration we take the coronal boundary as the object boundary and translate or rotate the axial and sagittal boundaries to match the coronal boundary. In a different approach from most strategies of registration based on the gray level images, our registration aims to adjust the boundary consisting of the 3D spatial coordinates. After registration, the boundaries in differently-oriented planes would share the same crossing points.

**Fig 4:**
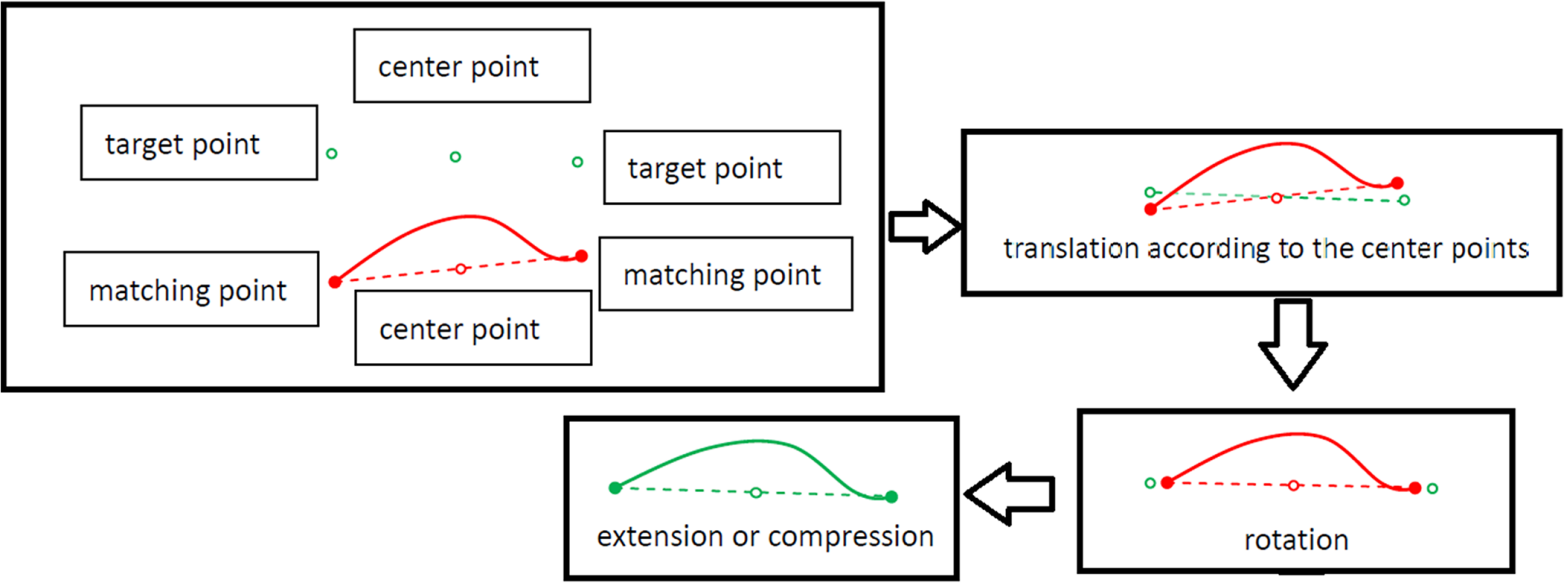
The process of registration.

A Matlab program code was written according to the registration algorithm. As shown in Fig 4, the coronal boundaries of the nasal cavity are cut by the axial plane. The target points are the crossing points lying in the coronal section that are cut by the axial plane. Similarly, the matched points are the crossing points lying in the axial section cut by the coronal plane. The overall boundary points in the axial section between the two adjacent matched points are translated, rotated and linearly stretched such that the piece of the axial boundary with two endpoints (the two adjacent matched points) intersects with the coronal boundary at the two corresponding target points. In this way, all the axial boundaries can be corrected piecewise based on the coronal section.

**Fig 5:**
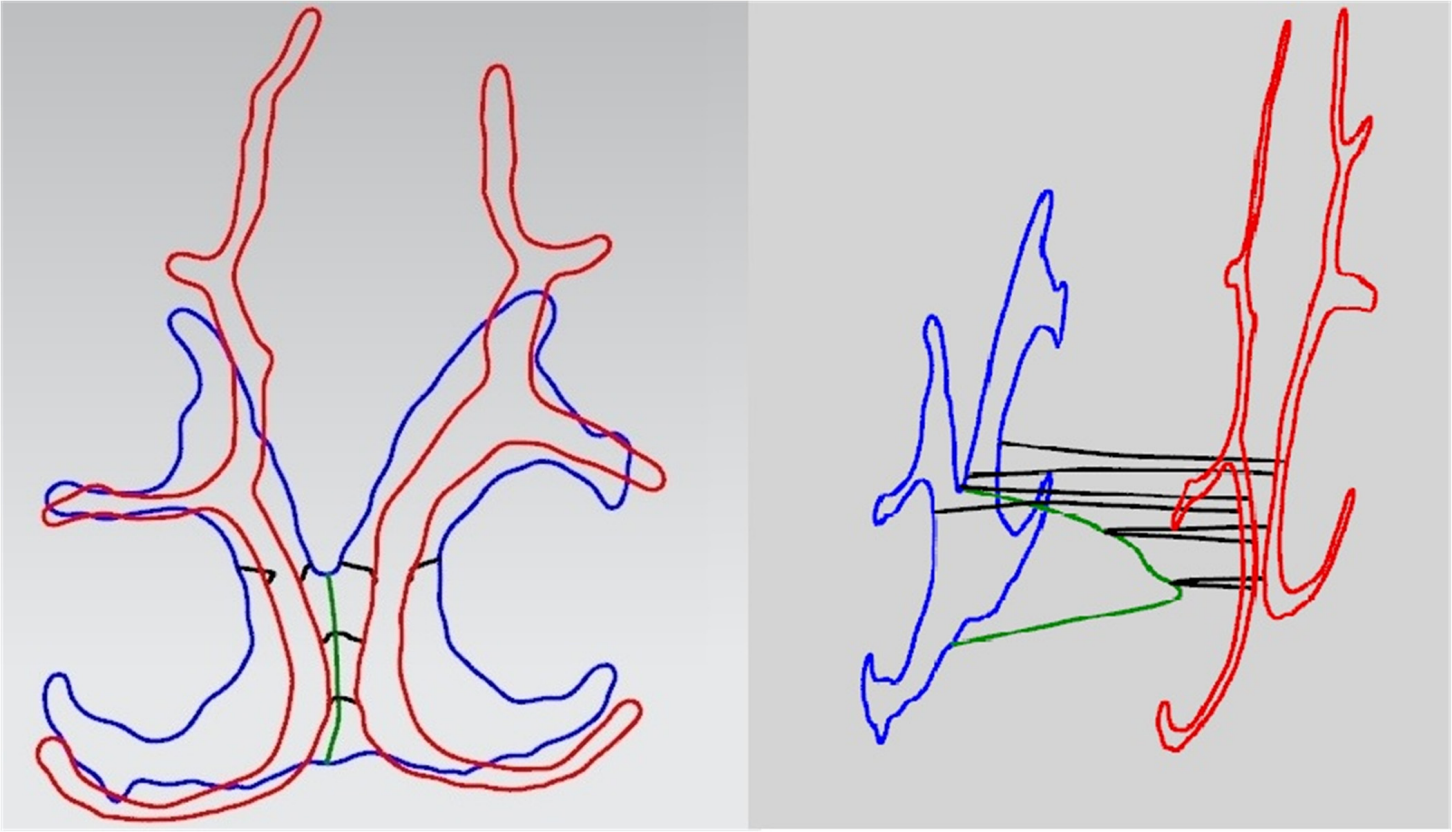
After registration, the boundaries of the nasopharynx sharing the same crossing points are shown. The left panel is viewed from the front, and the right one is the lateral view. For the right panel, the distance between the two coronal sections was enlarged artificially to clearly show the registered curves between the two sections.

For registration, a key step is to determine the location of the posterior border of the nasal septum in the coronal plane. The posterior nasal septum terminates at the anterior nasopharynx where the confluence of the left and right nasal cavities is located. Fig 5 shows that, for the last few coronal boundaries, the left and right nasal cavity gradually merge and form the nasopharynx. For the two neighboring coronal images the number of airways changes from two to one. One can judge from the anatomy of the nasal cavity that the posterior border of the nasal septum terminates between the two neighboring coronal slices, and is also the boundary separating the nasal cavity and the nasopharynx. Because of the spacing distance between two adjacent slices of coronal images, the posterior border of the nasal septum cannot be completely captured by the coronal images. The arbitrariness exists for the boundary of the nasal septum if only the coronal images are used to reconstruct the nasal cavity. If only these images are used, the location of the anterior wall of the nasopharynx will be undetermined, and the shape and volume of the nasopharynx will be approximately built in some 3D reconstruction methods. Such a structure cannot accurately reflect the real anatomical features. To determine the correct position of the anterior wall of the nasopharynx, we use the boundary information from both the related axial and sagittal boundaries. We make use of the axial images that can show the full position of the nasal septum to compensate for the shortage of coronal images in the coronal plane. Also, the sagittal boundary of the nasal septum can make up for the loss of axial images. It is necessary to register the posterior boundaries of the nasal septum (or the anterior wall of the nasopharynx) in different planes. First, the posterior border of the nasal septum in the axial plane was adjusted to meet the boundary in the coronal plane. Then, the sagittal boundary of the nasal septum was also modified to cross with the boundaries in the coronal and axial planes.

The last registration deals with the boundaries of the oropharynx, which mainly consists of the axial boundary constrained by the sagittal boundary. Unlike the nasal cavity, which is enclosed by bone and cartilage, the oropharynx is surrounded by soft tissue. Thus, the shape and position of the wall of the airway will change in the process of respiration. The axial image is sensitive to the movement of the head in the scanning plane. This kind of motion should be calibrated by the constraints of the sagittal boundary, and the amount of displacement of the axial boundary can be evaluated by the gap between the axial boundary and the sagittal boundary. Then, we modulate the axial boundaries according to the value of the displacement. After this step, the sagittal boundary was calibrated according to the crossing points of the axial and sagittal planes as in the case of the nasal cavity. Finally, both the axial and sagittal boundaries were registered.

The three sets of images belonging to differently-oriented planes were registered following the steps described above. All the boundaries were unified to a whole frame. The segmented boundaries in different planes are like the parts of an elephant touched by blind people; the goal of registration is to combine the parts in a reasonable way to form an integrated picture. In this respect, more complete anatomical information is provided compared with only making use of one set of images in the same scanning plane.

### Reconstruction

Three steps were taken to reconstruct the 3D structure of the UA. First, the left and right nasal cavities were constructed using the NX UG software (Siemens). The next step was to build the oropharynx. Last, the part connecting the nasal cavity and the oropharynx was constructed.

To construct the NURBS surface, the points in the same slice were connected to form the NURBS curve. In the reconstruction process, one important issue is to order the points on all boundaries such that the points in one slice of the image can correspond to the adjacent points in other slices. We reordered all the boundary points according to the anatomical features of the nasal cavity, i.e., the points consisting of inferior turbinate in one slice should be connected to the points of inferior turbinate in other slices. This step was completed by the Matlab program that was written to select the characteristic points satisfying the curvature conditions (local maxima or minima). Before the construction, all the points in the coronal plane were divided into three main parts: inferior turbinate, middle turbinate and super turbinate. From the first slice to the last slice, the points belonging to the same part were arranged in the same group, which was to form the boundary of the given region.

Then, all the NURBS curves in the coronal plane sweep to form the nasal cavity according to the characteristic points. The oropharynx was built based on the axial boundaries as shown in Fig 3, and was constrained by the sagittal boundaries. The transition part from the nasal cavity to the nasopharynx was constructed based on the coronal boundaries that were constrained by both the axial and sagittal boundaries.

## Results

**Fig 6:**
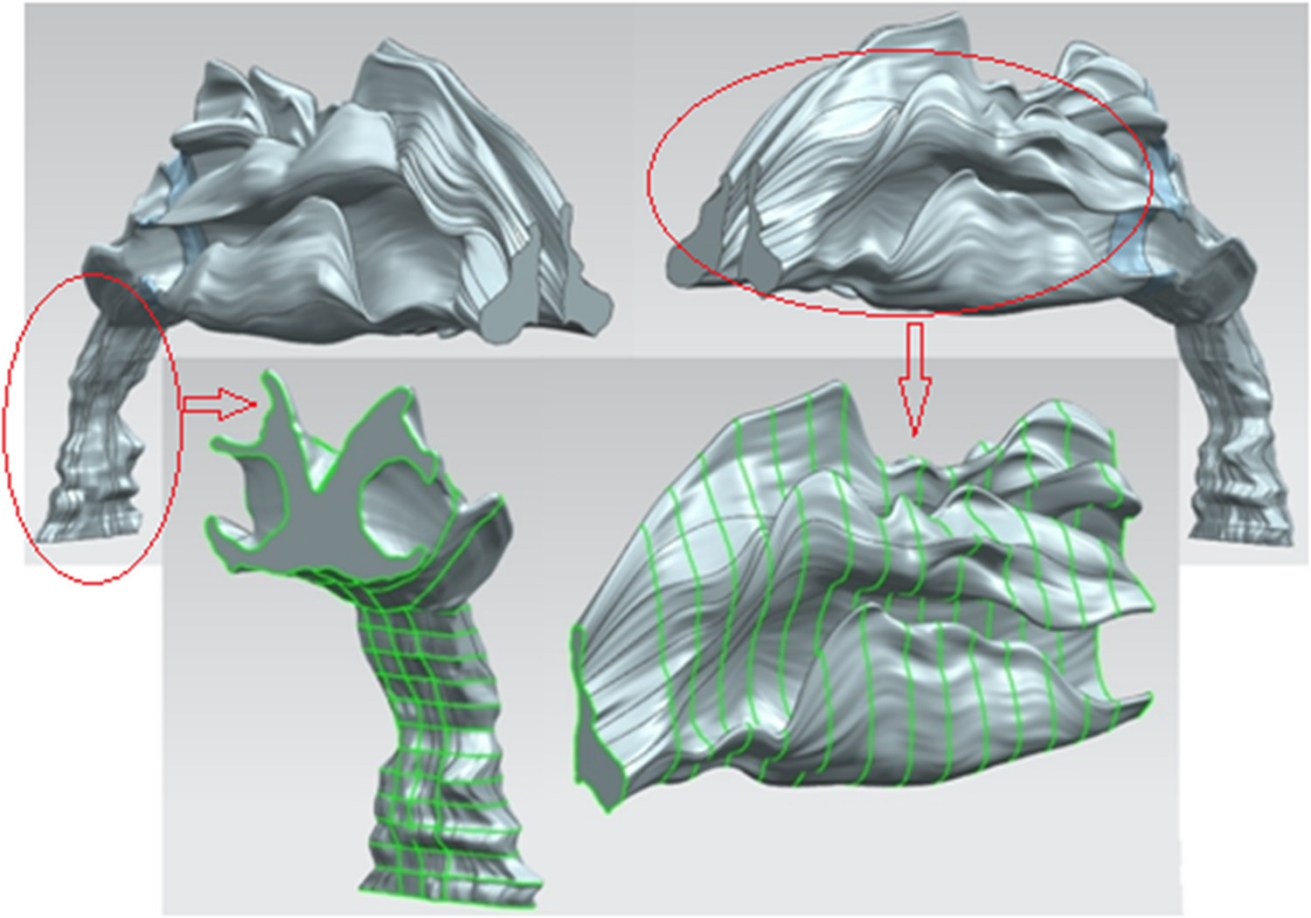
The 3D upper airway constructed based on the NURBS surface using NX UG software.

**Fig 7:**
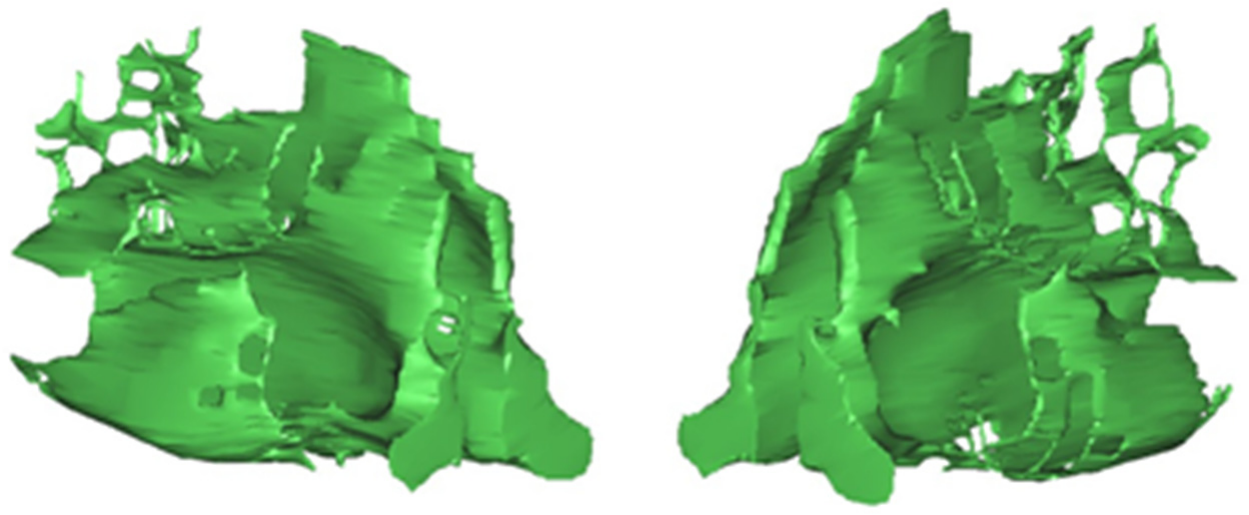
The 3D nasal cavity constructed using Mimics. This reconstruction is based on the same boundaries segmented by the level set method.

Fig 6 shows the 3D upper airway reconstructed using the NX UG commercial software. Fig 7 is the same nasal cavity constructed using Mimics. The segmented coronal boundaries were converted to binary images, with the inner region of the boundary kept at a fixed lower gray value and the outer region at a fixed higher gray value in the Mimics models. Compared with the construction results given by NX UG, there were many small holes in the Mimics models, and therefore much work needs to be done to fill these holes in the 3D nasal cavity structure. Furthermore, to produce smooth geometry, the constructed object needs to be meshed by the specific function of the software. The more steps that are taken to smooth the constructed structure, the more the constructed object deviates from the real structure. In our construction, the constructed 3D UA was surrounded by the surfaces that passed through all the boundaries, and the region between the adjacent boundaries was constrained by the registered boundary lines crossing all the target boundaries. These procedures can make the reconstructed object depict the real anatomical structure.

**Fig 8:**
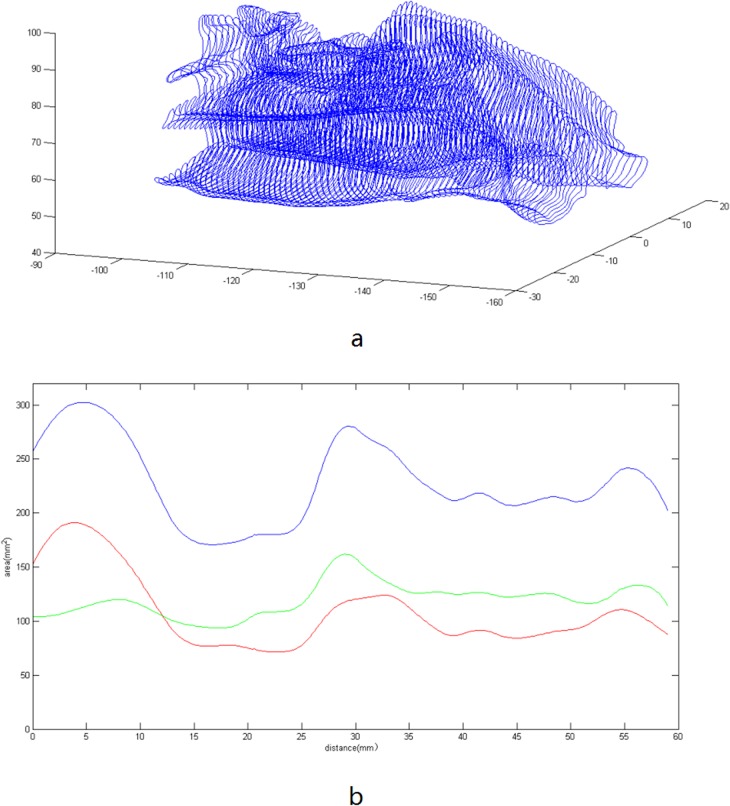
The boundary curves and the area of the nasal cavity. a) The coronal section curves from the nasal cavity. The distance between the neighboring curves is 0.2mm. b) The area of the nasal cavity in the coronal plane enclosed by the section curves, where the blue line is the total area of the left and right nasal cavity, and the green and red lines correspond to the areas of the right and left nasal cavity, respectively.

From the reconstructed 3D model of the UA, some useful anatomical information can be obtained, such as the volume of the UA and the narrowest region of the nasal cavity, which are important factors for evaluating the nasal resistance [31]. The curves of the area for the section of the nasal cavity in the coronal plane are shown in Fig 8. The position of the smallest section area can also be found from the area curves that are also shown in Fig 8. By integral calculation, the volume of the right nasal cavity was 7085.9mm^3^, and the left was 6398.2mm^3^.

## Discussion

In this paper, we developed an optimized scheme to reconstruct the upper airway based on MRI. A realistic structure is an essential requirement for simulating the flow in the UA. One can build finite element models on a well reconstructed geometry of the upper airway to analyze the flow behavior. Because the flow pattern is closely related to the complex structure of the upper airway, to accurately reflect the real flow behavior, the geometry of the upper airway must be carefully constructed. To date, the most accurate way to segment tissue is manual operation. However, that takes much effort and time. Although the method of automatic segmentation is time-saving, it often fails to deal with complex boundaries. It is worth considering the time consumption and ensuring the accuracy of the result at the same time. Proper manual intervention is necessary to realize this purpose. To segment the nasal cavity, we manually chose the initial region for the level set method. As pointed out, the manually chosen initial region ensures the quality of the segmentation. The level set method is widely used in medical image segmentation, and we extend this method to tackle the upper airway successfully, especially in the segmentation of the nasal cavity. The results indicate that manual intervention strengthens the power of the level set method.

To evaluate the accuracy of the adopted method of image segmentation, a conventional way is to compare automatic segmentation with manual segmentation, and to check the correspondence between them. For our segmentation, we combine the manual selection of the original region of the object with the level set method. This combination makes use of the accuracy of manual operation and the effectiveness of the level method, thus ensuring the quality of the final result.

For the reconstruction, we use NX UG commercial software to convert the 2D boundaries to the 3D NURBS surface. This is a different approach to that used by the popular medical image processing software Mimics, in which one must mesh the body of reconstruction to reduce irregularities to produce smooth geometry. One disadvantage of the mesh operation is its uncertainty. Too many mesh operations might make the reconstructed object deviate from the real shape. In our reconstruction process, there is no need to mesh the reconstructed body. The reconstructed NURBS surface passes through all the segmented 2D boundaries. Thus, the NURBS surface can well depict the shape of the UA. Furthermore, we use the MRI images in three scanning planes (axial, sagittal and coronal) to restore the anatomical structure by the process of registration. Compared with the conventional method that only uses one image direction, our results can more accurately reflect the anatomical characteristics. Our strategy of registration is based on the boundary coordinates, which can exactly produce the merging of boundaries in the three orthogonal planes.

We reconstruct the nasal cavity and the airway based on the NURBS surface with the bottom-up method. Our work is different from the UA model in [32], where the author used the commercial software Mimics to reconstruct the geometry of the UA based on CT images and converted the 3D boundary of the UA into the cloud points to reconstruct the structure using NURBS. Our process reduces the uncertainty of modeling from the smooth operation of Mimics software. Also, the NURBS surface was used to depict the geometry of the UA directly without the middle process of transmitting the constructed geometry into cloud points. It should be noted that the NURBS surface has been widely used in industry to design the geometry of products as an industrial standard for CAD (computer-aided design). Thus, our model is convenient for much post-processing, such as the surgical design of an OSA patient, the finite element computation of airflow dynamics in the UA, and the rapid prototyping of a model in vitro.

At the current stage, the method developed in this study may be more applicable in research, but it can also have many potential clinical applications. Our modeling can be used to evaluate various anatomical parameters of OSA patients accurately. OSA threatens people’s health, which can result in neurocognitive and cardiovascular problems [33]. The abnormality of the anatomical structure of UA is closely related to OSA. For example, a narrower and longer UA is more easy to collapse that can lead to OSA [34]. An accurately generated structure of the UA is critical to evaluate the risk factors of OSA, Many conclusions concerning OSA are based on 2D images of the UA [35]. The 3D UA can provide integrated anatomical information to quantify the anatomical characteristics related to OSA. In this paper, we provide a complete scheme to reconstruct a patient-specific 3D UA model, which can capture the anatomical features of UA accurately. This reconstruction can also be used to evaluate the success of sleep apnea oral appliances, or be used as a tool for the surgical planning of OSA [36–39]. In the recent years, numerical models based on realistic anatomical structures of the upper airway are developed to simulate in great detail the air flow and pressure distribution, and to predict the airway collapse and obstruction under different conditions in OSA studies [40, 41]. Our method developed in this study can provide an important tool for building 3D computational models of the upper airway for OSA studies. In addition, our result in Fig 8 can also help to evaluate the validation of acoustic rhinometry, which is widely used for preoperative diagnosis and decision-making about surgery of the nasal cavity [42].

In future work, different imaging methods and the reconstruction methods of the UA should be evaluated. Yet until now, we still lack information about the comparison between different imaging methods, such as MRI and CT, as well as the reconstruction methods. In consideration of this fact, the degree of the uncertainty between different methods is unclear. We hope that further research is able to solve this problem. With our work in improving 3D reconstruction, this present scheme will help to develop better patient-specific reconstructions of anatomical structures.
